# Supplementary material for: Higher energy and safer sodium ion batteries via an electrochemically made disordered Na3V2(PO4)2F3 material
Source: Nat Commun. 2019 Feb 4;10:585. doi: 10.1038/s41467-019-08359-y (PMC6362244; doi:10.1038/s41467-019-08359-y)
Supplement: Supplementary file 1 — Supplementary Information [file 41467_2019_8359_MOESM1_ESM.docx]

**Supplementary information**

Higher energy and safer sodium ion batteries via an electrochemically made disordered Na_3_V_2_(PO_4_)_2_F_3_ material

Yan *et al.*


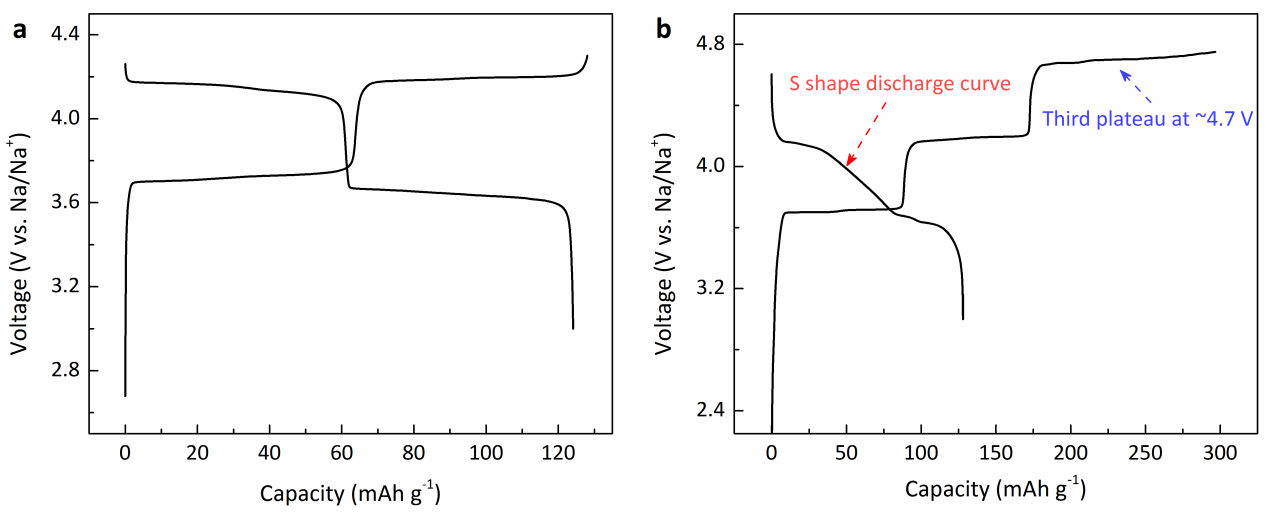


**Supplementary Figure 1**. The first charge-discharge curves of NVPF/Na cell cycled with 1 M NaPF_6_ dissolved in PC electrolyte between 4.3–3.0 V at C/10 rate (a); and cycled with 1 M NaPF_6_ dissolved in PC/EC/DMC (1/1/1 in volume ratio) electrolyte between 4.8 V to 3.0 V at C/10 rate (b).


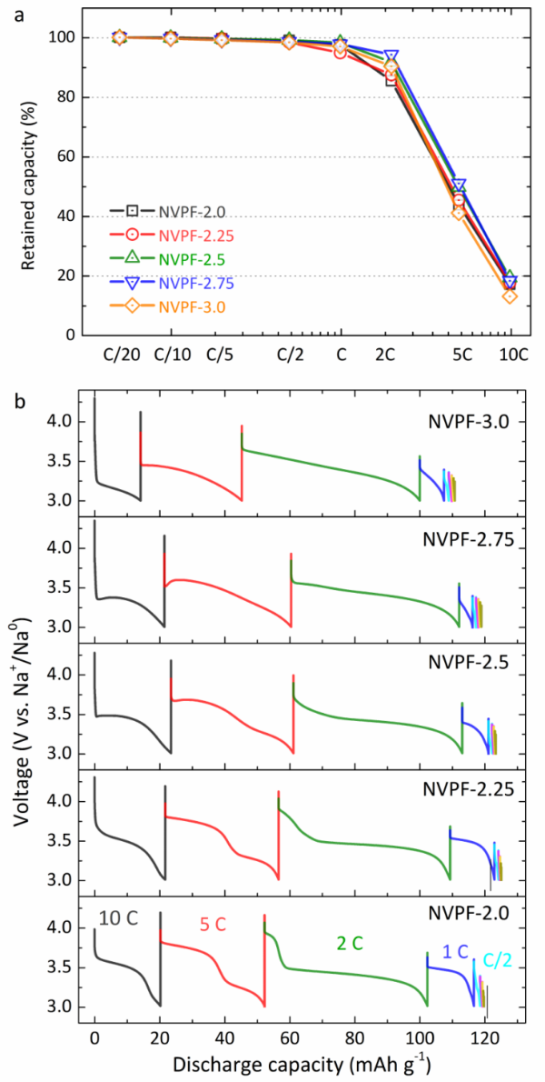


**Supplementary Figure 2**. The rate capability of NVPF-2.0, -2.25, -2.5, -2.75 and -3.0 samples when the discharge voltage was limited to 3.0 V (vs. Na+/Na0) at 10 C, 5 C, 2 C, C, C/2, C/5, C/10, and C/20 (C = 128 mA g^−1^) (a); and the corresponding signature curves (b).


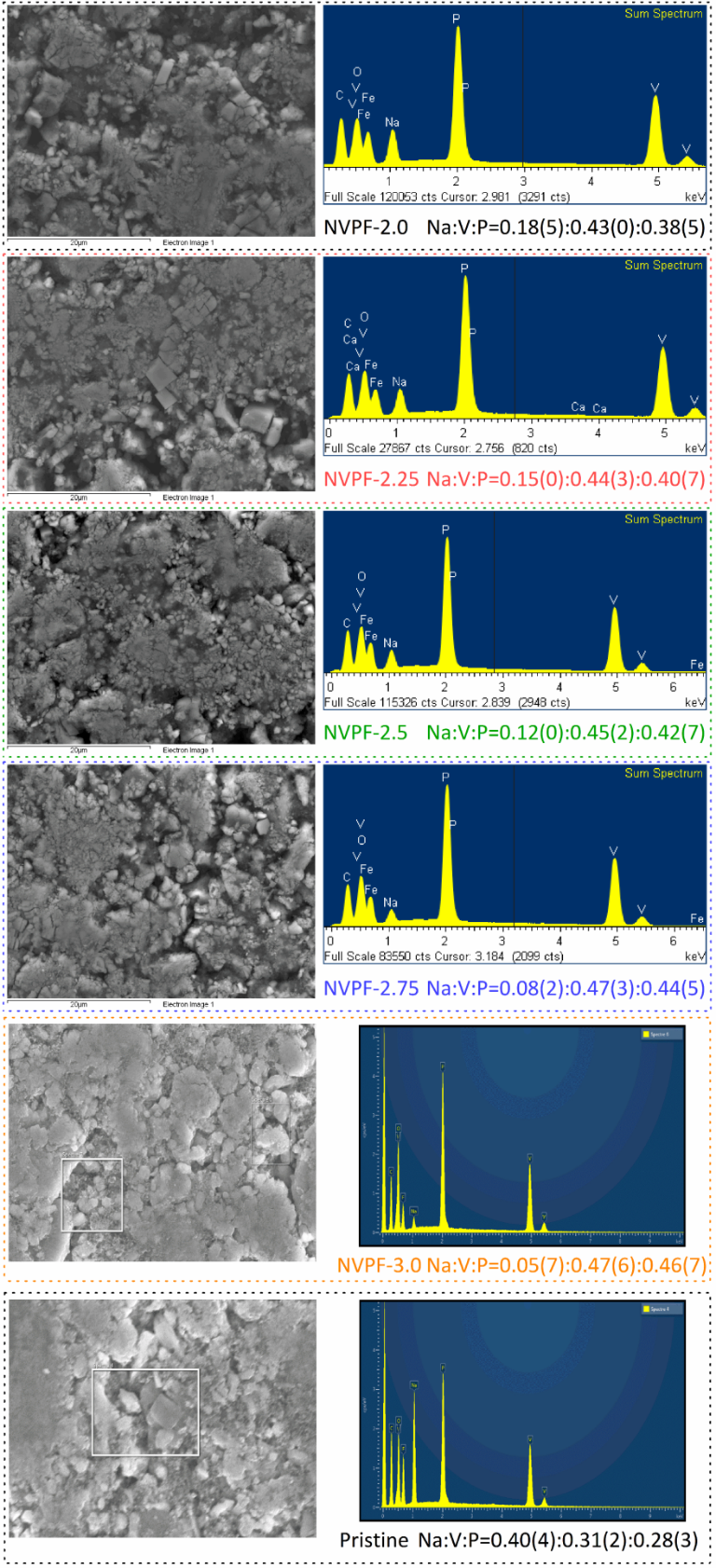


**Supplementary Figure 3**. SEM images and EDX results of charged-NVPF 2.0, NVPF-2.25, NVPF-2.5, NVPF-2.75, NVPF-3.0 and pristine samples.

**
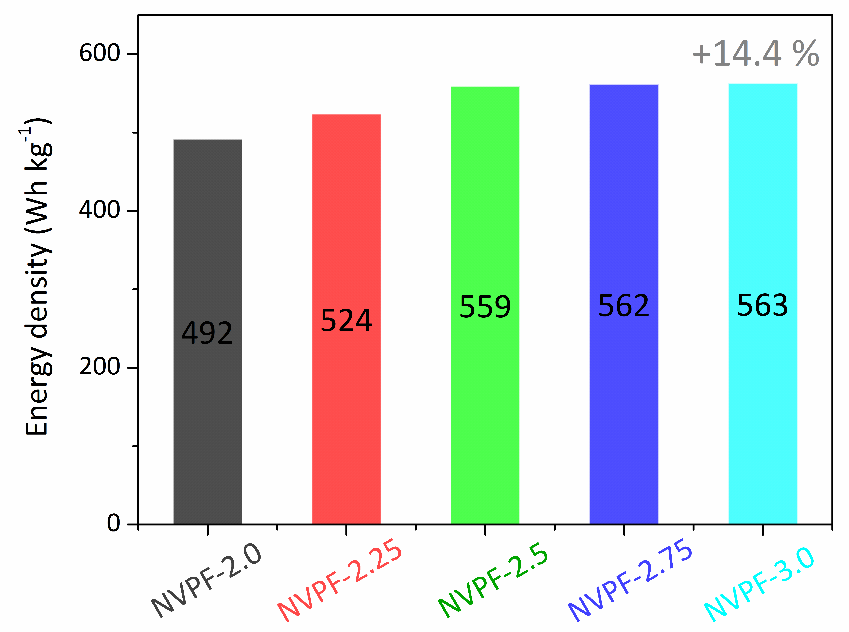
**

**Supplementary Figure 4**. The energy density of NVPF-2.0, NVPF-2.25, NVPF-2.5, NVPF-2.75 and NVPF-3.0 samples based on product of the discharge capacity and its voltage when the NVPF/Na cells were discharged to 1.0 V.


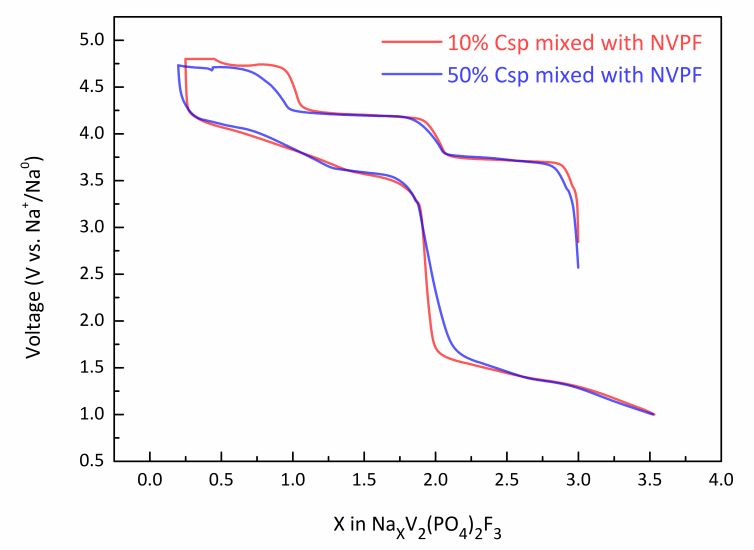


**Supplementary Figure 5**. The first charge-discharge curves of NVPF mixed with 10 % and 50 % Csp when the extracted Na ions in the first charge process was limited to 2.75 Na^+^ (Δx = 2.75); the current is C/10 (1 C = 128 mA g^–1^).


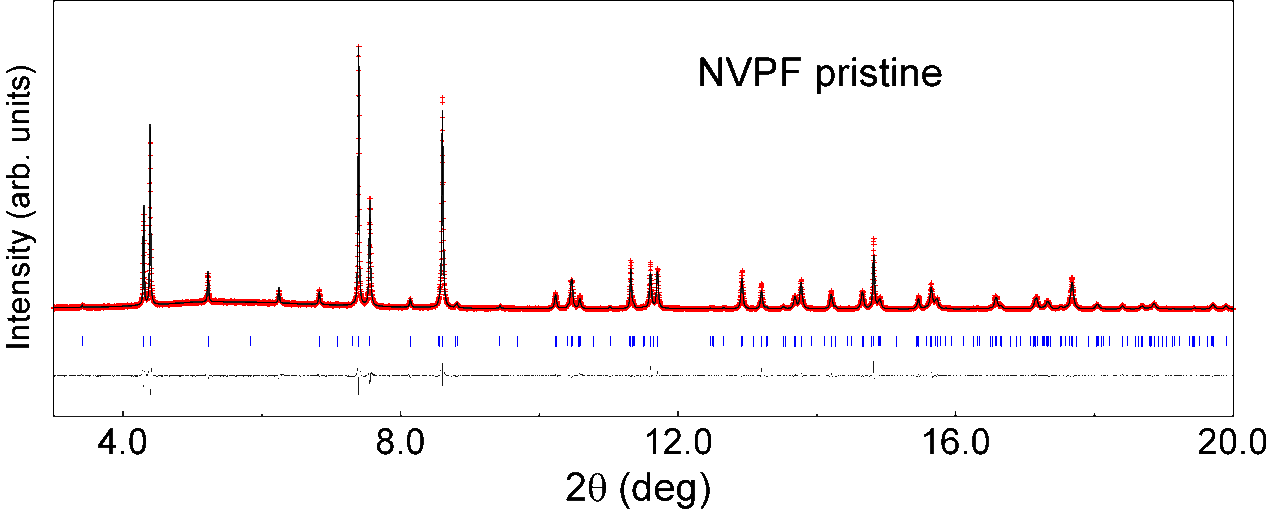


**Supplementary Figure 6**. Rietveld refinement of the synchrotron X-ray diffraction pattern of pristine Na_3_V_2_(PO_4_)_2_F_3_ (λ = 0.412 Ǻ). The red crosses, black continuous line and bottom green line represent the observed, calculated, and difference patterns, respectively. Vertical blue tick bars mark the reflection positions in the *Amam* space group.


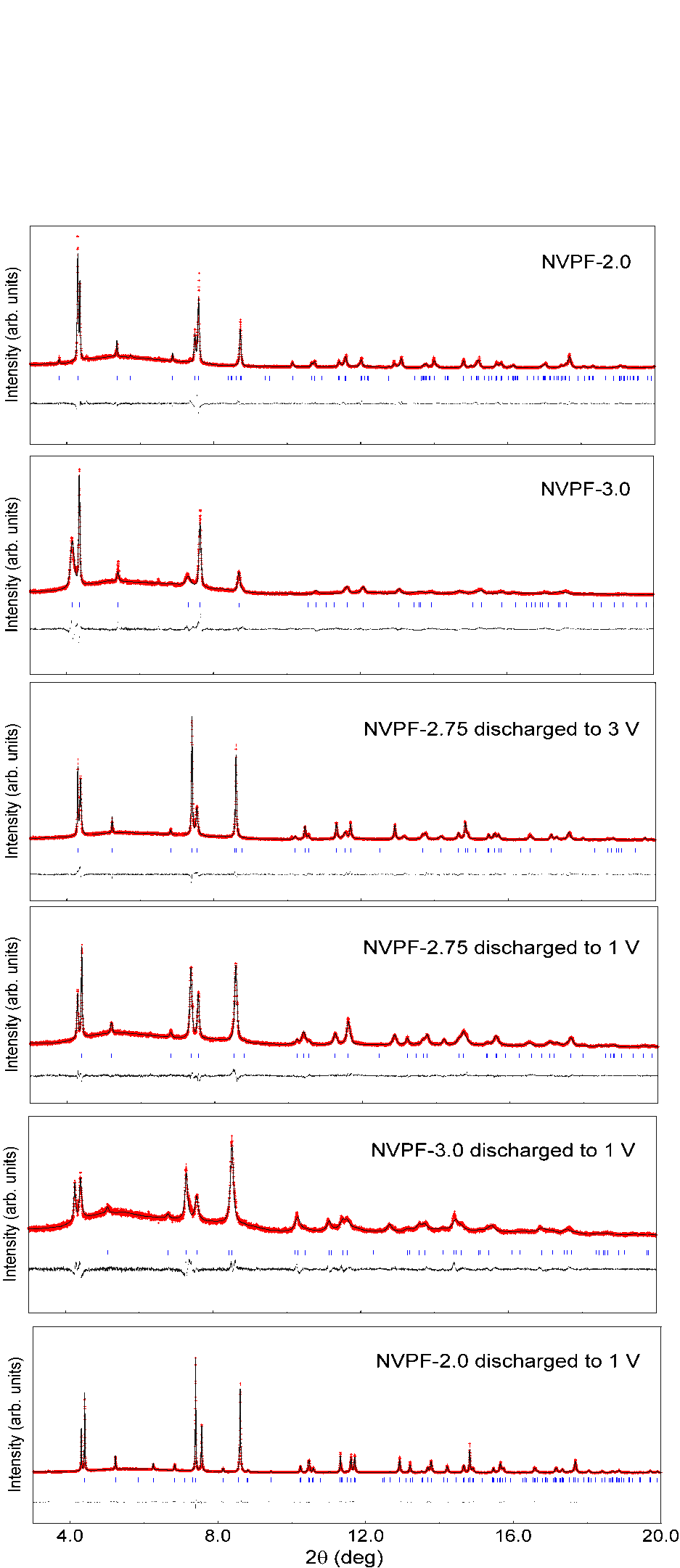


**Supplementary Figure 7**. Rietveld refinement of the synchrotron X-ray diffraction patterns of Na*_x_*V_2_(PO_4_)_2_F_3_ samples (λ = 0.412 Ǻ). The red crosses, black continuous line and bottom green line represent the observed, calculated, and difference patterns, respectively. Vertical blue tick bars mark the reflection positions.


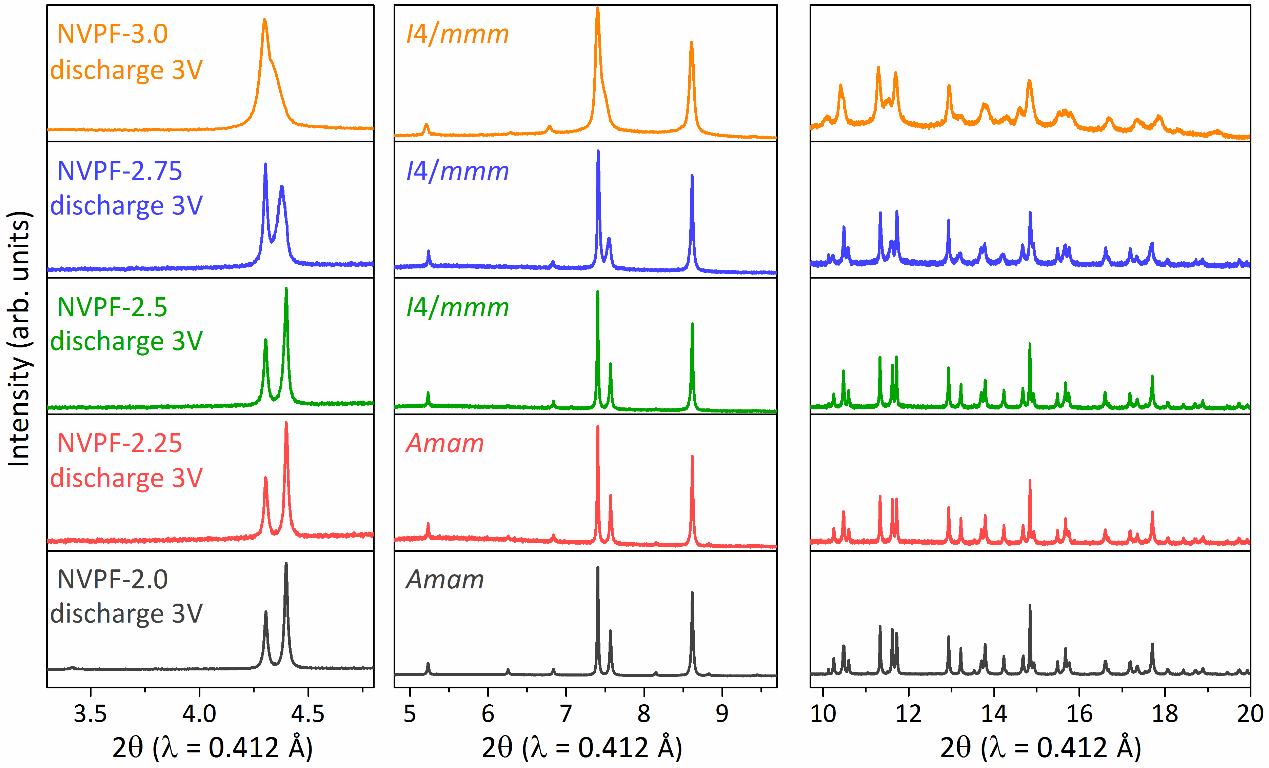


**Supplementary Figure 8**. 11BM XRD patterns of NVPF-3.0, NVPF-2.75, NVPF-2.5, NVPF-2.25, NVPF-2.0 samples discharged to 3.0 V.


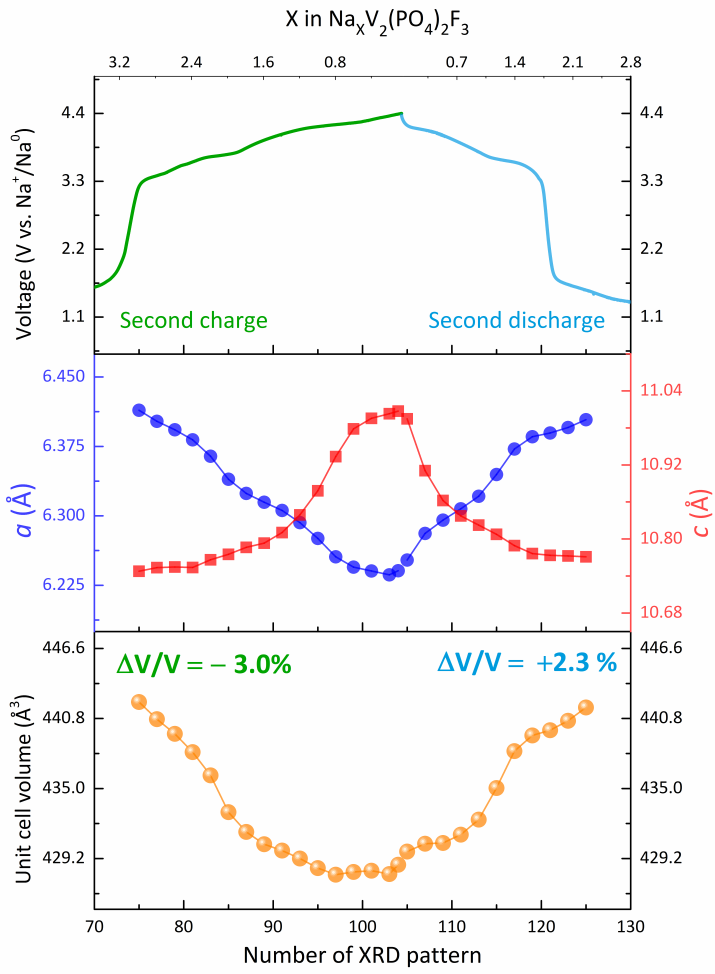


**Supplementary Figure 9**. Lattice parameters evolution deduced from the refinement of XRD data collected on cycling using an *in situ* XRD cell.


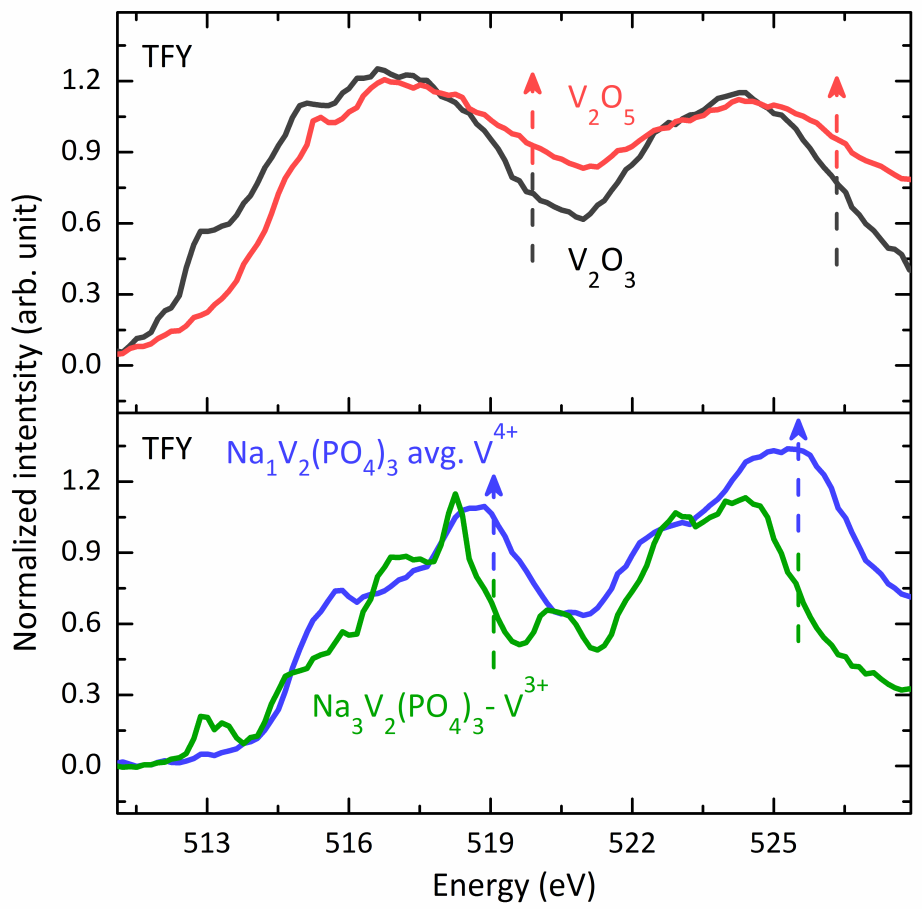


**Supplementary Figure 10**. The normalized V L-edge X-ray absorption spectra of various reference samples obtained in Total Fluorescence Yield (TFY) mode.


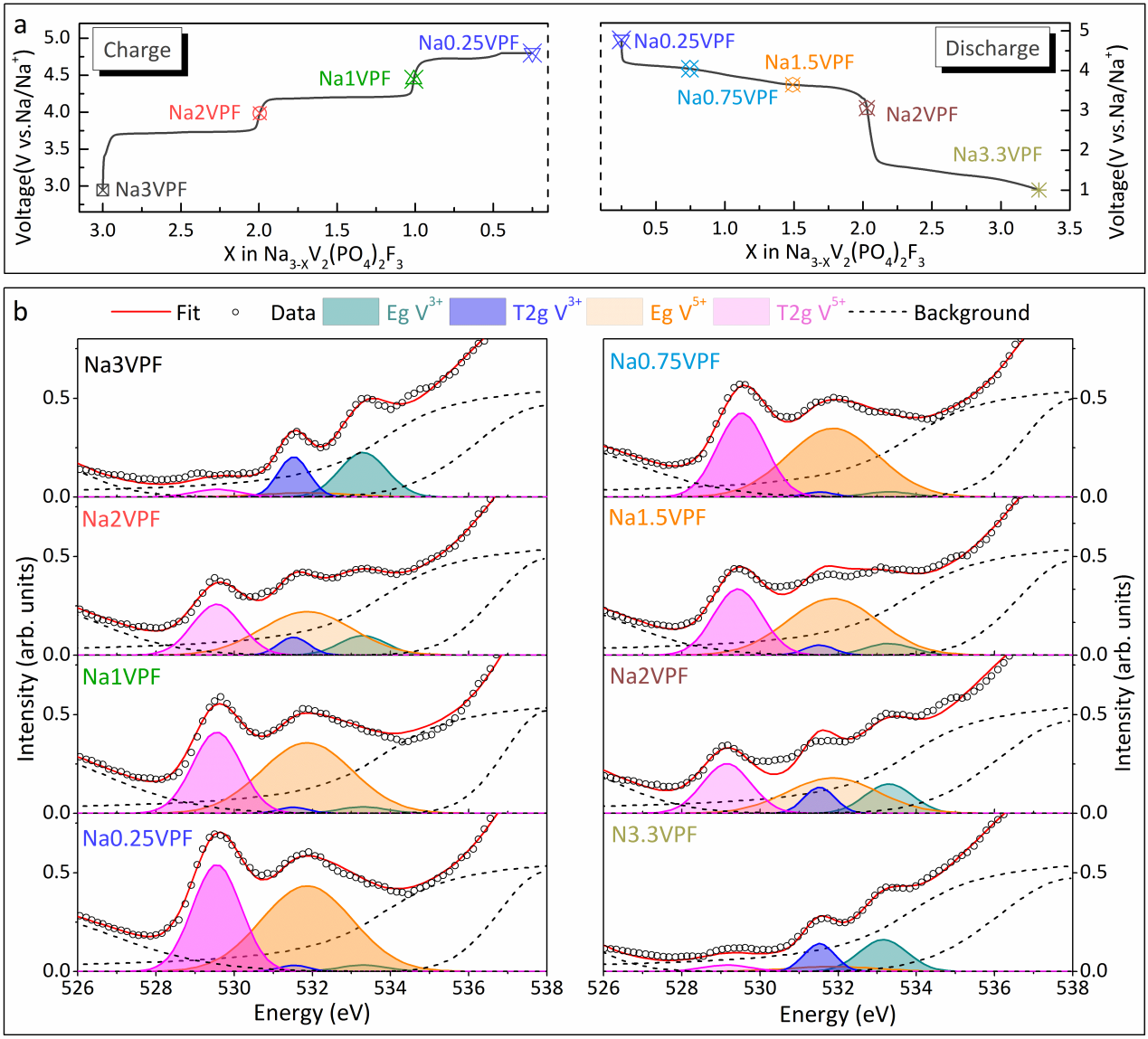


**Supplementary Figure 11**. The voltage-composition curve of NVPF-2.75 sample with asterisk labeled (a), the pre edge of O K edge spectra and its corresponding fitting data of Na_3_V_2_(PO_4_)_2_F_3_ (Na3VPF), Na_2_V_2_(PO_4_)_2_F_3_ (Na2VPF), Na_1_V_2_(PO_4_)_2_F_3_ (Na1VPF), Na_0_V_2_(PO_4_)_2_F_3_ (Na0VPF), Na_0.75_V_2_(PO_4_)_2_F_3_ (Na0.75VPF), Na_1.5_V_2_(PO_4_)_2_F_3_ (Na1.5VPF), Na_2_V_2_(PO_4_)_2_F_3_ (Na2VPF), Na_3.3_V_2_(PO_4_)_2_F_3_ (Na3.3VPF) samples (b).


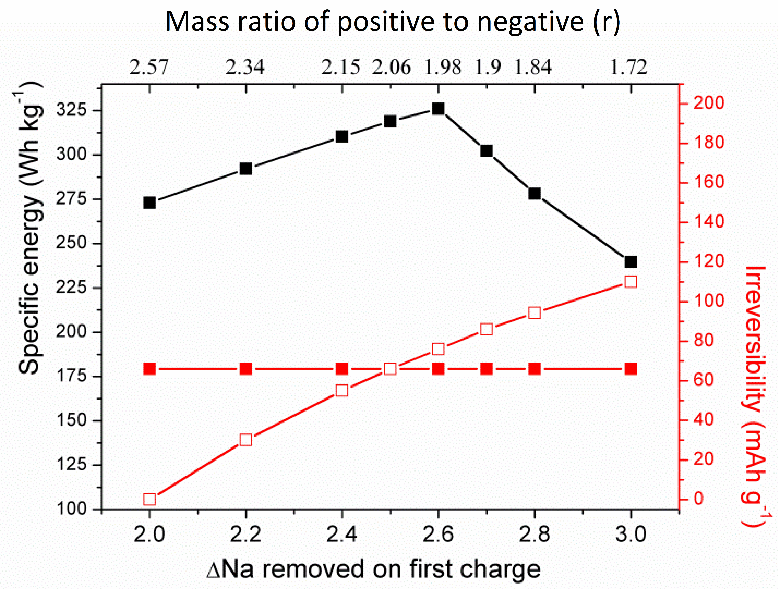


**Supplementary Figure 12**. The variation of the cell energy (based on the total mass of NVPF and C) in full Na-ion coin cells cycled from 4.3 to 2 V as a function of key parameters of mass ratio of positive to negative electrode and the amount of sodium removal on first charge. The specific energy is calculated for the total mass of positive and negative active materials and the irreversibility on first cycle is mentioned for the weight of hard carbon electrode.


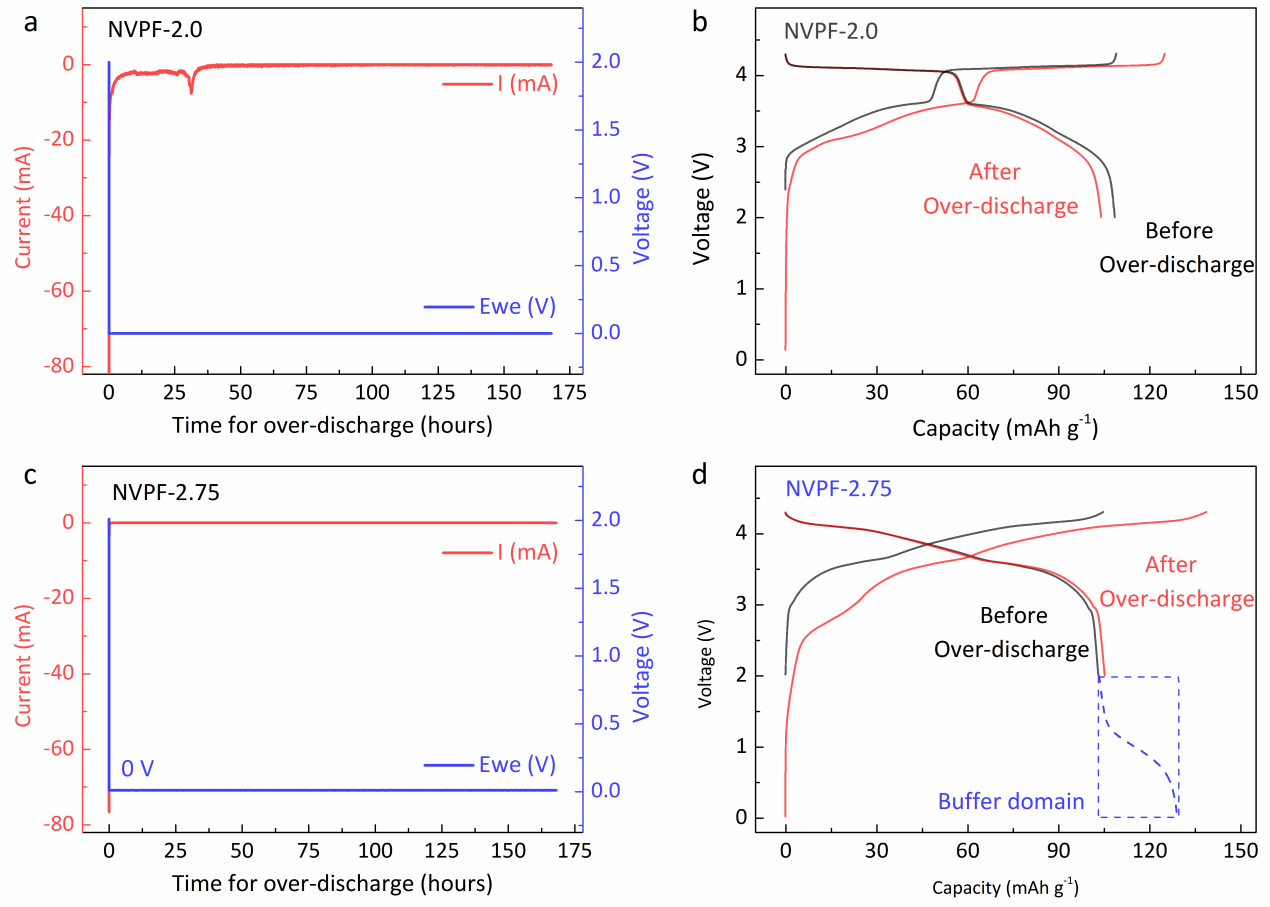


**Supplementary Figure 13**. The voltage and current monitored during the zero volts over-discharge tests of NVPF-2.0 (a) and NVPF-2.75 sample (c); and the voltage-capacity curves before and after zero volts over-discharge tests of NVPF-2.0 (b) and NVPF-2.75 sample (d).

**Supplementary Table 1** The energy density of related polyanionic compounds from literature and our work

| Sample | Chemical formula | Energy density (Wh kg^–1^) | Reference |
| --- | --- | --- | --- |
| 1 | Na_3_V_2_O_2_(PO_4_)_2_F | 388 | ^1^ |
| 2 | Na_3_V_2_O_2_*_x_*(PO_4_)_2_F_3-2_*_x_* | 323 | ^2^ |
| 3 | Na_3_(VOPO_4_)_2_F | 447 | ^3^ |
| 4 | Na_3_(VPO_4_)_2_F_3_ | 461 | ^3^ |
| 5 | Na_3_(VOPO_4_)_2_F | 421 | ^4^ |
| 6 | Na_3_V_2_(PO_4_)_2_O_2_F | 486 | ^5^ |
| 7 | Na_3_V_2_(PO_4_)_2_FO_2_ | 540 | ^6^ |
| 8 | Na_3_V_2_(PO_4_)_2_F_2.5_O_0.5_ | 434 | ^7^ |
| 9 | Na_4_MnV(PO_4_)_3_ | 400 | ^8^ |
| 10 | Na_3_V_2_(PO_4_)_2_O_1.6_F_1.4_ | 518 | ^9^ |
| 11 | Na_3_V_2_(PO_4_)_2_F_3_ | 563 | Our work |

**Supplementary Table 2** Structural parameters of NVPF-2.0 (chemical composition Na_1_V_2_(PO_4_)_2_F_3_) determined from the Rietveld refinement of the synchrotron 11BM XRD data.

| Na_1_V_2_(PO_4_)_2_F_3_, *Cmc*2_1_ space group,  *a* = 8.81577(19) Å, *b* = 8.8288(3) Å, *c* = 11.00215(16) Å,  *V* = 856.32(3) Å^3^, *V*/Z = 214.08 Å^3^, *R*_Bragg_ = 7.01 %, χ^2^ = 1.18 | | | | | | |
| --- | --- | --- | --- | --- | --- | --- |
| **atom** | **Wyckoff site** | **x** | **y** | **Z** | **B (Å^2^)** | **Occ** |
| V1 | 4*a* | 0.5 | 0.249(2) | 0.3380(2) | 0.40(6) | 1 |
| V2 | 4*a* | 0 | 0.254(2) | 0.1897(4) | 0.40(6) | 1 |
| P | 8*b* | 0.2500(19) | 0.4980(18) | 0.2581(10) | 0.32(9) | 1 |
| O1 | 8*b* | 0.347(2) | 0.118(3) | 0.357(2) | 0.16(12) | 1 |
| O2 | 8*b* | 0.347(3) | 0.410(3) | 0.370(2) | 0.16(12) | 1 |
| O3 | 8*b* | 0.151(3) | 0.090(4) | 0.188(3) | 0.16(12) | 1 |
| O4 | 8*b* | 0.149(3) | 0.413(4) | 0.193(3) | 0.16(12) | 1 |
| F1 | 4*a* | 0.5 | 0.261(5) | 0.520(3) | 0.4(2) | 1 |
| F2 | 4*a* | 0.5 | 0.275(4) | 0.8736(12) | 0.4(2) | 1 |
| F3 | 4*a* | 0.5 | 0.231(5) | 0.1925(16) | 0.4(2) | 1 |
| Na1 | 4*a* | 0.5 | 0.0572(17) | 0.011(5) | 8.0(5) | 1 |

**Supplementary Table 3** Structural parameters of NVPF-3.0 (approximate composition Na_0_V_2_(PO_4_)_2_F_3_) determined from the Rietveld refinement of the synchrotron 11BM XRD data.

| Na_0_V_2_(PO_4_)_2_F_3_, *I*4*/mmm* space group,  *a* = 6.19887(18) Å, *c* = 11.3865(8) Å,  *V* = 437.53(3) Å^3^, *V*/Z = 218.76 Å^3^_,_ *R*_Bragg_ = 10.6 %, χ^2^ = 1.34 | | | | | | |
| --- | --- | --- | --- | --- | --- | --- |
| **atom** | **Wyckoff site** | **x** | **y** | **z** | **B (Å^2^)** | **Occ** |
| P1 | 4*d* | 0 | 0.5 | 0.25 | 8.3(3) | 1 |
| V1 | 4*e* | 0 | 0 | 0.1683(3) | 1.83(10) | 1 |
| O1 | 16*n* | 0 | 0.3080(7) | 0.1520(4) | 7.2(3) | 1 |
| F2 | 4*e* | 0 | 0 | 0.6893(4) | 6.3(3) | 1 |
| F1 | 2*a* | 0 | 0 | 0 | 6.3(3) | 1 |

**Supplementary Table 4** Structural parameters of NVPF-2.75 discharged to 3 V determined from the Rietveld refinement of the synchrotron 11BM XRD data.

| Na_x_V_2_(PO_4_)_2_F_3_ (x = 2.40(1) from refinement), *I*4*/mmm* space group,  *a* = 6.39489(5) Å, *c* = 10.81238(14) Å,  *V* = 442.17(1) Å^3^, *V*/Z = 221.08 Å^3^, *R*_Bragg_ = 6.6 %, χ^2^ = 1.11 | | | | | | |
| --- | --- | --- | --- | --- | --- | --- |
| **atom** | **Wyckoff site** | **x** | **y** | **z** | **B (Å^2^)** | **Occ** |
| V1 | 4*d* | 0 | 0 | 0.18306(12) | 1.30(4) | 1 |
| P1 | 4*e* | 0 | 0.5 | 0.25 | 1.51(5) | 1 |
| O1 | 16*n* | 0 | 0.3081(3) | 0.16411(19) | 1.46(5) | 1 |
| F2 | 4*e* | 0 | 0 | 0.3615(3) | 2.35(5) | 1 |
| F1 | 2*a* | 0 | 0 | 0 | 2.35(5) | 1 |
| Na1 | 8*h* | 0.2804(6) | 0.2804(6) | 0 | 1.60(18) | 0.36(2) |
| Na2 | 16*l* | 0.4099(17) | 0.2190(18) | 0 | 1.60(18) | 0.12(2) |

**Supplementary Table 5** Structural parameters of NVPF-2.75 discharged to 1 V determined from the Rietveld refinement of the synchrotron 11BM XRD data.

| Na_x_V_2_(PO_4_)_2_F_3_ (x = 3.25(1) from refinement), *I*4*/mmm* space group,  *a* = 6.43624(10) Å, *c* = 10.7602(2) Å,  *V* = 445.74(2) Å^3^, *V*/Z = 222.87 Å^3^, *R*_Bragg_ = 7.55 %, χ^2^ = 1.03 | | | | | | |
| --- | --- | --- | --- | --- | --- | --- |
| **atom** | **Wyckoff site** | **x** | **y** | **z** | **B (Å^2^)** | **Occ** |
| V1 | 4*d* | 0 | 0 | 0.18440(13) | 0.78(3) | 1 |
| P1 | 4*e* | 0 | 0.5 | 0.25 | 1.62(6) | 1 |
| O1 | 16*n* | 0 | 0.3119(3) | 0.16399(19) | 1.80(7) | 1 |
| F2 | 4*e* | 0 | 0 | 0.3663(4) | 1.97(8) | 1 |
| F1 | 2*a* | 0 | 0 | 0 | 1.97(8) | 1 |
| Na1 | 8*h* | 0.2735(8) | 0.2735(8) | 0 | 3.7(2) | 0.6(1) |
| Na2 | 16*l* | 0.416(4) | 0.231(3) | 0 | 3.7(2) | 0.10(2) |

**Supplementary Table 6** Structural parameters of NVPF-3 discharged to 1 V determined from the Rietveld refinement of the synchrotron 11BM XRD data.

| Na_x_V_2_(PO_4_)_2_F_3_ (x = 3.36(1) from refinement), *I*4/*mmm* space group,  a = 6.5080(3) Å, c = 10.7233(7) Å,  V = 454.17(4) Å^3^, V/Z = 227.08 Å^3^, R_Bragg_ = 6.18 %, χ2 = 1.03 | | | | | | |
| --- | --- | --- | --- | --- | --- | --- |
| **atom** | **Wyckoff site** | **x** | **y** | **z** | **B (Å^2^)** | **Occ** |
| V1 | 4*d* | 0 | 0 | 0.1897(3) | 1.42(5) | 1 |
| P1 | 4*e* | 0 | 0.5 | 0.25 | 3.61(15) | 1 |
| O1 | 16*n* | 0 | 0.3194(7) | 0.1644(4) | 3.14(14) | 1 |
| F2 | 4*e* | 0 | 0 | 0.3736(9) | 6.2(2) | 1 |
| F1 | 2*a* | 0 | 0 | 0 | 6.2(2) | 1 |
| Na1 | 8*h* | 0.2698(5) | 0.2698(5) | 0 | 2.9(2) | 0.83(1) |
| Na2 | 16*l* | 0.416 | 0.231 | 0 | 2.9(2) | 0.01(2) |

**Supplementary Table 7** Structural parameters of NVPF-2.0 discharged to 1 V determined from the Rietveld refinement of the synchrotron 11BM XRD data.

| Na_x_V_2_(PO_4_)_2_F_3_ (x = 3.03(1) from refinement), *Amam* space group,  *a* = 9.04235(5) Å, *b* = 9.06005(5) Å, *c* = 10.76963(4) Å,  *V* = 882.29 (2) Å^3^, *V*/Z = 220.57 Å^3^, *R*_Bragg_ = 4.39 %, χ^2^ = 1.01 | | | | | | |
| --- | --- | --- | --- | --- | --- | --- |
| **atom** | **Wyckoff site** | **x** | **y** | **z** | **B (Å^2^)** | **Occ** |
| V1 | 8*g* | 0.25 | 0.25254(20) | 0.18389(5) | 0.181(12) | 1 |
| P | 8*e* | 0 | 0 | 0.2458(3) | 0.324(18) | 1 |
| O1 | 16*h* | 0.0968(3) | 0.0939(4) | 0.1632(3) | 0.41(2) | 1 |
| O2 | 16*h* | 0.0908(3) | 0.3983(4) | 0.1658(3) | 0.41(2) | 1 |
| F1 | 4*c* | 0.25 | 0.2533(7) | 0 | 0.56(3) | 1 |
| F2 | 8*g* | 0.25 | 0.7609(5) | 0.13197(15) | 0.56(3) | 1 |
| Na1 | 4*c* | 0.75 | 0.0211(6) | 0 | 1.68(7) | 0.917(4) |
| Na2 | 8*f* | 0.5398(4) | 0.2861(3) | 0 | 1.68(7) | 0.764(6) |
| Na3 | 8*f* | -0.1335(8) | 0.4118(8) | 0 | 1.68(7) | 0.294(4) |

**Supplementary Table 8** The intensity of the pre-edge signals of the O K-edge peak ascribed to V^3+^ (t_2g_, e_g_) and V^5+^ (t_2g_, e_g_) during the charge process of NVPF-2.75 sample.

| Peak intensity | Na_0.75_V_2_(PO_4_)_2_F_3_ | Na_1.5_V_2_(PO_4_)_2_F_3_ | Na_2_V_2_(PO_4_)_2_F_3_ | Na_3.3_V_2_(PO_4_)_2_F_3_ |
| --- | --- | --- | --- | --- |
| V^3+^ (t_2g_) | 0.03 | 0.05 | 0.13 | 0.14 |
| V^3+^ (e_g_) | 0.05 | 0.09 | 0.23 | 0.24 |
| V^5+^ (t_2g_) | 0.66 | 0.52 | 0.39 | 0.05 |
| V^5+^ (e_g_) | 1.01 | 0.83 | 0.52 | 0.07 |

**Supplementary Table 9** The intensity of the pre-edge signals of the O K-edge peak ascribed to V^3+^ (t_2g_, e_g_) and V^5+^ (t_2g_, e_g_) during the discharge process of NVPF-2.75 sample.

| Peak intensity | Na_0.75_V_2_(PO_4_)_2_F_3_ | Na_1.5_V_2_(PO_4_)_2_F_3_ | Na_2_V_2_(PO_4_)_2_F_3_ | Na_3.3_V_2_(PO_4_)_2_F_3_ |
| --- | --- | --- | --- | --- |
| V^3+^ (t_2g_) | 0.03 | 0.05 | 0.13 | 0.14 |
| V^3+^ (e_g_) | 0.05 | 0.09 | 0.23 | 0.24 |
| V^5+^ (t_2g_) | 0.66 | 0.52 | 0.39 | 0.05 |
| V^5+^ (e_g_) | 1.01 | 0.83 | 0.52 | 0.07 |

**Supplementary Table 10** The electrochemical data of NVPF/C full cells derived from Supplementary Figure 12 with various mass ratio.

| Mass ratio (+/-) | Na extracted in the first charge | Irreversibility of the NVPF positive electrode (mAh g^−1^) | Irreversible capacity of Carbon negative electrode (mAh g^−1^) | Energy density based on the total mass of NVPF and C (Wh kg^−1^) |
| --- | --- | --- | --- | --- |
| 2.57 | 2.0 | 24.4 | 66 | 273 |
| 2.34 | 2.2 | 30.2 | 66 | 292 |
| 2.15 | 2.4 | 55.2 | 66 | 310 |
| 2.06 | 2.5 | 66.4 | 66 | 319 |
| 1.98 | 2.6 | 76.2 | 66 | 326 |
| 1.9 | 2.7 | 86.2 | 66 | 302 |
| 1.84 | 2.8 | 94.5 | 66 | 278 |
| 1.72 | 3.0 | 110.2 | 66 | 240 |

**Supplementary Note 1**

The ^23^Na MAS-NMR spectrum of pristine NVPF (Fig. 6a) shows a broad peak centered at 116 ppm, resulting from the Fermi contact interaction due to the presence of paramagnetic V^3+^ ions. Though there are three different sodium sites (Na1, Na2 and Na3) in the pristine NVPF, they are in very similar environments in terms of vanadium cations and therefore similar contact shifts, as shown by previous NMR measurements and DFT calculations.^10^ Equally, the ^31^P NMR spectrum of pristine NVPF (Fig. 6f) consists in one broad peak with 2 spinning sidebands (black stars) around 6320 ppm, which corresponds to the phosphorus site in the Amam structure and accounts for 83% of the overall intensity. The additional small peak (red arrow) with two sidebands (red stars) around 4730 ppm, may correspond to a phosphate unit adjacent to one V^4+^ and three V^3+^, indicating that nearly 4% of the vanadium are in the V^4+^ state in the pristine sample^11^.

On charge, the NMR spectra of NVPF-2.5 sample confirms the oxidation of V^3+^ with reduced Fermi contact shifts for ^23^Na and ^31^P (Fig. 6b,g)^12^. More specifically, there is a depletion of the single ^23^Na environment upon charging consistent with the presence of a single Na site within the Cmc2_1_ structure. The corresponding ^31^P spectrum (Fig. 6g) is broad indicating the distribution of vanadium oxidation states between 4+ and 5+ inside the structures (Cmc2_1_ and I4/mmm) observed in the NVPF-2.5 sample. Interestingly, a very narrow peak (around 3% of the total intensity) is observed for ^31^P around 0 ppm, potentially correspond to phosphate groups surrounded by diamagnetic V^5+^ species (black dashed line on the right hand side). Their presence is expected for an average state of V^4.25+^, as detected in by XAS (vide infra), or as in recent studies combined with ^51^V NMR. However, it is ten times more intense than predicted from a random distribution of V^5+^ ions (i.e. 0.25^4^ = 0.3%). A similar phenomenon was observed in previous studies on NVPF_3−_*_x_*O*_x_*, where the statistical analysis showed that the phosphate groups appeared to be more surrounded by oxidized vanadium than expected^12^, possibly indicating that upon oxidation, electron hoping may be triggered, averaging of the paramagnetic interactions with the ^31^P spins.

The NVPF-3 sample on discharge to 3.0V is expected to be in the I4/mmm structure with one phosphorus site and two partially occupied sodium sites. The ^23^Na NMR spectrum (Fig. 6c) shows a broad peak, with a maximum around 115 ppm, and a long tail extending to −150 ppm, a signature of the local disorder^13^, as expected for a structure which presents two sodium sites with partial occupancy, associated with distributions of the neighboring vanadium oxidation states. For ^31^P (Fig. 6h), a broad, non-uniform distribution is observed, with two maxima: the first and smallest one is around 6300 ppm, with a signature similar to the NVPF-pristine ^31^P site (surrounded by 4 V^3+^), and the second one is around 1000-2000 ppm, with a large width indicating a variety of environments (V^4+^/V^3+^), associated eventually with electron hoping for the same reasons stated before. Once NVPF-3.0 is discharged to 1.0V, an additional environment is detected for ^23^Na (Fig. 6d), around -10 ppm (green dashed line) and accounting for 13% of the total intensity, which may be a signature of neighboring V^2+^ ions and/or from an SEI. The broad distribution observed for sodium environments is conserved, while it is notably absent from the spectrum recorded for NVPF-2.0 discharged to 1.0V (Fig. 6e) which conserved its original structure and the associated NMR signature (black dashed line), apart from a small contribution around 0 ppm. In the ^31^P NMR spectrum, a broad distribution of environments is observed around 4000 ppm with a reduced shift seemingly stemming from the Fermi contact with V^2+^ species (Fig. 6i), in stark contrast to the spectrum observed for NVPF-2.0 discharged to 1.0V (Fig. 6j), which is very similar to the spectrum recorded for the pristine NVPF (black dashed line on the left hand side). Interestingly, the signature of the original environments has disappeared, indicating that most phosphate groups may be relatively close to a V^2+^ ion.

**Supplementary Note 2**

Na_3_V_2_(PO_4_)_2_F_3_ (NVPF) cycled between 4.3–3.0 V using 1 M NaPF_6_ dissolved in PC electrolyte delivers two distinguished flat plateaus, which locate at ~3.7 V and 4.2 V indicating a biphasic process in each plateau as shown in Supplementary Figure 1a. During our previous electrolyte study^14^, we concluded that DMC can easily react with Na metal, forming soluble species that shuttle to the NVPF electrode to be oxidized. As shown in Supplementary Figure 1b, prolonged plateaus appear because of the oxidation soluble species, and the third plateau at ~4.7 V emerges when the charge voltage limit is increased to 4.8 V (vs. Na^+^/Na^0^) by using electrolyte of 1 M NaPF_6_ dissolved in PC/EC/DMC (1/1/1 in volume ratio). Though a huge irreversibility on first charge is observed which is partly due to the decomposition of electrolyte containing DMC, the discharge curve of NVPF electrode shows a clear ‘S’ shape transformation as compared to the NVPF cycled in 4.3-3.0 V, which suggests that the sodium is removed from the NVPF structure also at ~4.7 V plateau with an irreversible structural change.

After a certain extraction of Na ions (Δx = 2.0, 2.25, 2.5, 2.75, 3.0) in the first charge process, the rate capability of *in situ* electrochemically formed NVPF-2.0, NVPF-2.25, NVPF-2.5, NVPF-2.75 and NVPF-3.0 was measured by the signature curves discharged down to 3.0 V (vs. Na^+^/Na^0^) as shown in Supplementary Figure 2. The capacity retention ratios at various current rates (10C, 5C, 2C, C, C/2, C/5, C/10, C/20) coincide within these five samples (Supplementary Figure 2a), indicating that the created new ‘NVPF’ could deliver a competitive power rate performance. The discharge capacities of NVPF-2.25, -2.5, -2.75 samples are even slightly higher than NVPF 2.0 at high current density (2 C, 5 C, and 10 C), as shown in Supplementary Figure 2b.

After 1^st^ charging with specific amount of Na^+^ (Δx = 2.0, 2.25, 2.5, 2.75, 3.0) extracted, the NVPF electrodes were washed by DMC three times before SEM- EDX tests. Supplementary Figure 3 shows the SEM images and EDX spectra of the NVPF-2.0, NVPF-2.25, NVPF-2.5, NVPF-2.75, NVPF-3.0 charged and pristine samples. Obviously, the area of the Na peak decreases with increasing the amount of Na^+^ extracted from NVPF electrodes. Note that the exact O and F concentrations cannot be reliably quantified from EDX, therefore we have taken the Na:V:P atomic ratio of each sample to determine the formula of the NVPF charged samples. Specifically, on the basis of EDX data, NVPF-2.0, -2.25, -2.5, and -2.75 show Na:V:P atomic ratios of 0.18(5):0.43(0):0.38(5), 0.15(0):0.44(3):0.40(7), 0.12(0):0.45(2):0.42(7), and 0.08(2):0.47(3):0.44(5) respectively. These results indicate that the formula of charged NVPF electrodes is close to Na_0.88_V_2_(PO_4_)_2_F_3_, Na_0.71_V_2_(PO_4_)_2_F_3_, Na_0.55_V_2_(PO_4_)_2_F_3_, and Na_0.31_V_2_(PO_4_)_2_F_3_, which agrees well with the Na_1_V_2_(PO_4_)_2_F_3_ (NVPF-2.0), Na_0.75_V_2_(PO_4_)_2_F_3_ (NVPF-2.25), Na_0.5_V_2_(PO_4_)_2_F_3_ (NVPF-2.5), Na_0.25_V_2_(PO_4_)_2_F_3_ (NVPF -2.75). However, for the charged NVPF-3.0 sample, the Na:V:P atomic ratio is 0.05(7):0.47(6):0.46(7) corresponding a formula of Na_0.21_V_2_(PO_4_)_2_F_3_, which means that there are some electrolyte oxidation when extracting the remaining 0.25 Na ions by potentiostatic charging at 4.8 V (vs. Na^+^/Na^0^).

Supplementary Figure 4 shows the discharge energy density of NVPF-2.0, NVPF-2.25, NVPF-2.5, NVPF-2.75 and NVPF-3.0 samples. Although the capacity increases from ~140 to 200 mAh g^-1^ for the NVPF-3.0 samples as compared to NVPF-2.0 sample, the energy density only increases by ~15 % because of the low plateau around 1.6-1.0 V (vs. Na^+^/Na^0^).

To explore the effect of the conductive agent on the kinetics of NVPF, 10 % and 50 % Csp was mixed with NVPF in a powder state that was tested within in the Swagelok type cells. As shown in Supplementary Figure 5, the charge-discharge curves of the two samples neatly superimpose, indicating that the electronic conductivity is not the reason causing the voltage drop in the discharge process for NVPF-2.75 sample.

Supplementary Figure 8 shows the *ex situ* synchrotron XRD spectra of NVPF-2.0, NVPF-2.25, NVPF-2.5, NVPF-2.75, NVPF-3.0 samples when they were discharged to 3.0 V (vs. Na^+^/Na^0^). Although the NVPF-2.5 sample still maintains an *I*4/*mmm* structure when discharged to 3.0 V, it reverts back to its original *Amam* structure when discharged to 1.0 V (Fig. 4b). NVPF-2.25 and NVPF-2.0 samples keep the *Amam* structure when discharged to 3.0 V, while NVPF-2.75 and NVPF-3.0 remain in the *I*4/*mmm* structure.

After the 1^st^ cycle, the lattice parameters and cell volume of the NVPF-2.75 sample during the second charge and discharge were obtained from the *in situ* XRD tests as shown in Supplementary Figure 9. During the charge, the cell volume was decreased from 442.16 Å^3^ to 428.68 Å^3^, and then increases to 441.70 Å^3^. It corresponds to a volume decrease of -3.0% in the charge process, and a volume increase of +2.3% in the discharge process.

Supplementary Figure 10 shows the V L-edge XAS spectra of the reference samples obtained in Total Fluorescence Yield (TFY) mode. It consists in two peaks around 516 and 523 eV which can be attributed to the L2/L3 splitting. . Moving from V_2_O_3_ (V^3+^) to V_2_O_5_ (V^5+^) or from Na_3_V_2_(PO_4_)_3_ (V^3+^) to Na_1_V_2_(PO_4_)_3_ (V^4+^), these peaks shift to higher energy. Such evolution is ascribed to the oxidation of vanadium.

To better quantify the evolution of valence of Vanadium during the charge and discharge process, the pre edge of O K edge data was fitted as shown in Supplementary Figure 11. Four gaussian functions were considered for the e_g_ and t_2g_ of V^3+^ and V^5+^. Position and width of the gaussian were fixed to 531.5 eV and 0.39, 533.3 eV and 0.61, 529.6 eV and 0.62, and 531.9 eV and 1.16 for V^3+^ (e_g_), V^3+^ (t_2g_), V^5+^ (e_g_) and V^5+^ (t_2g_), respectively. Note that the width of the e_g_ is larger than the t_2g_ due to the larger band width of e_g_ over t_2g_ levels. The background was taken into account using an arctan function for the edge step, and two Gaussian functions for the V L2 edge and the O K edge white line. The detailed intensity of various samples is summarized in Supplementary Table 8 and 9. Based on the fitting data, we can conclude that the content of V^5+^ gradually increases while V^3+^ diminishes in the charge process, which shows the opposite trend during the discharge process.

To evaluate the practical aspects of increasing amount of Na^+^ removed from the 3^rd^ plateau, the specific energy is calculated by changing the positive to negative material weight ratio. Supplementary Figure 12 and Supplementary Table 10 shows the variation of the cell specific energy in full Na-ion coin cells cycled from 4.3 to 2 V as a function of key parameters such as i) mass ratio of positive to negative electrode, ii) the amount of removed Na (Δx) while fixing the reversible capacity of the negative electrode at 250 mAh g^−1^. It appears that the best composition capacity-wise is for Δx = 2.6; a composition which falls into the Na removal range for which there is the formation of the disordered NVPF phase. Worth mentioning is that such maximum can varies depending upon the nature of the C electrode that controls the SEI., the greater the SEI formation, the greatest the shift towards Δx = 3. Although capacitive-wise, there is no incentive to push the Na removal beyond 2.6 (except with poor carbons), an advantage of using Δx > 2.5 is to have Na-ion cells that can be easily and safely handled in their discharged state (0 V) while minimizing the penalty to be paid in terms of energy density.

To evaluate the over-discharge protection function originated from the low voltage plateau in newly formed NVPF 2.75 sample, we performed an over-discharge test at 0 V by chronoamperometry method. Supplementary Figure 13a and 13c show the current and voltage during the 0 V over-discharge for NVPF-2.0 and NVPF-2.75 samples. The NVPF-2.75 cells soon reached at a steady current close to 0 mA, while NVPF-2.0 shows a fluctuation that may rooted in the side reactions. Comparing the charge-discharge curves before and after 0 V over-discharge tests for NVPF-2.0 (Supplementary Figure 13b) and NVPF-2.75 (Supplementary Figure 13d), we can conclude that the discharge capacity didn’t decrease because of the buffer region in the NVPF-2.75 sample.

**Supplementary Methods**

**Electrochemical tests**

The power rate of NVPF-2.0, NVPF-2.25, NVPF-2.5, NVPF-2.75, and NVPF-3.0 samples were determined by signature curves as described below^15^. The cells were first charged to extract certain amount of Na ions (Δx), and then the signature curves were measured by decreasing the current from 10C to C/20 (1C = 128 mA g^−1^) with limited condition by relaxing for 10 hours or |dV|/dt < 6 mV/h before switching to another current rate. Both of the NVPF samples discharged to 3 V and 1 V (vs. Na^+^/Na^0^) were measured.

The over-discharge tests were described as follows. The samples of NVPF-2.0 or NVPF-2.75 in the full cells were firstly cycled for 5 cycles, and then the cells were forced to 0 V for 1 week using chronoamperometry technique. After over-discharge tests, the cells were cycled again. All of the electrochemical tests were performed with a potentiostat (Biologic, France)

**SEM-EDX measurement**

After extracting a certain amount of Na ions during the 1^st^ charge in the NVPF/Na Swagelok cells, the SEM images and EDX spectra of NVPF-2.0, -2.25, -2.5, -2.75, -3.0 and pristine samples were collected by using a FEI Magellan 400 SEM equipped with a monochromated electron column.

**In-situ XRD measurement**

In-situ XRD studies were performed by using a home designed stainless Swagelok type cell with a Be window^16^, where an ultrathin Al foil was placed between the powder and Be window to avoid the oxidation of Be window. The XRD patterns were obtained by using a Cu-K radiation source (λ_1_ = 1.54056 Å, λ_2_ = 1.54439 Å) with a LynxEye detector when the cell was cycling.

**Supplementary References**

1. Xu, M., *et al*. Theoretical and Experimental Study of Vanadium-Based Fluorophosphate Cathodes for Rechargeable Batteries. *Chem. Mater.* **26**, 3089–3097 (2014).

2. Kumar, P.R., Jung Y.H., Lim, C.H., & Kim, D.K.. Na_3_V_2_O_2x_(PO_4_)_2_F_3-2x_: a stable and high-voltage cathode material for aqueous sodium-ion batteries with high energy density. *J. Mater. Chem. A* **3**, 6271–6275 (2015).

3. Zhao, J.M., *et al*. A phase-transfer assisted solvo-thermal strategy for low-temperature synthesis of Na_3_(VO_1-x_PO_4_)_2_F_1+2x_ cathodes for sodium-ion batteries. *Chem. Commun.* **51**, 7160–7163 (2015).

4. Qi, Y.R., *et al*. Superior Na-Storage Performance of Low-Temperature-Synthesized Na_3_(VO_1-x_PO_4_)_2_F_1+2x_ (0 ≤x ≤ 1) Nanoparticles for Na-Ion Batteries. *Angew. Chem. Int. Edit.* **54**, 9911–9916 (2015).

5. Guo, J.-Z.*, et al.* High-Energy/Power and Low-Temperature Cathode for Sodium-Ion Batteries: In Situ XRD Study and Superior Full-Cell Performance. *Adv. Mater.* **29**, 1701968 (2017).

6. Bianchini, M., Xiao, P., Wang, Y., & Ceder, G. Additional Sodium Insertion into Polyanionic Cathodes for Higher‐Energy Na‐Ion Batteries. *Adv. Energy Mater.* **7**, 1700514 (2017).

7. Broux, T.*, et al.* Temperature Dependence of Structural and Transport Properties for Na_3_V_2_(PO_4_)_2_F_3_ and Na_3_V_2_(PO_4_)_2_F_2.5_O_0.5_. *Chem. Mater.* **30**, 358–365 (2018).

8. Chen, F.*, et al.* A NASICON-Type Positive Electrode for Na Batteries with High Energy Density: Na_4_MnV(PO_4_)_3_. *Small Methods* 1800218 (2018).

9. Li, C.*, et al.* High-energy nanostructured Na_3_V_2_(PO_4_)_2_O_1.6_F_1.4_ cathodes for sodium-ion batteries and a new insight into their redox chemistry. *J. Mater. Chem. A* **6**, 8340–8348 (2018).

10. Park, Y-U*, et al.* Tailoring a fluorophosphate as a novel 4 V cathode for lithium-ion batteries. *Sci. Rep.* **2**, 704 (2012).

11. Broux, T.*, et al.* Strong Impact of the Oxygen Content in Na_3_V_2_(PO_4_)_2_F_3-_*_y_*O_y_ (0≤y≤0.5) on Its Structural and Electrochemical Properties. *Chem. Mater.* **28**, 7683–7692 (2016).

12. Dacek, S.T., Richards, W.D., Kitchaev, D.A., & Ceder, G. Structure and Dynamics of Fluorophosphate Na-Ion Battery Cathodes. *Chem. Mater.* **28**, 5450–5460 (2016).

13. Massiot, D.*, et al.* Topological, Geometric, and Chemical Order in Materials: Insights from Solid-State NMR. *Accounts Chem. Res.* **46**, 1975–1984 (2013).

14. Yan, G., *et al*. Assessment of the Electrochemical Stability of Carbonate-Based Electrolytes in Na-Ion Batteries. *J. Electrochem. Soc.* **165**, A1222–A1230 (2018).

15. Doyle, M., Newman, J., & Reimers, J. A quick method of measuring the capacity versus discharge rate for a dual lithium-ion insertion cell undergoing cycling. *J. Power Sources* **52**, 211–216 (1994).

16. Morcrette, M.*, et al.* In situ X-ray diffraction techniques as a powerful tool to study battery electrode materials. *Electrochim. Acta* **47**, 3137–3149 (2002).
